# Organic acids mitigate *Streptococcus agalactiae* virulence in Tilapia fish gut primary cells and in a gut infection model

**DOI:** 10.1186/s13620-024-00272-1

**Published:** 2024-05-27

**Authors:** Petculescu Ciochina Liliana, Gabi Dumitrescu, David McCleery, Ioan Pet, Tiberiu Iancu, Lavinia Stef, Nicolae Corcionivoschi, Igori Balta

**Affiliations:** 1 Faculty of Bioengineering of Animal Resources, University of Life Sciences King Mihai I from Timisoara, Timisoara, 300645 Romania; 2 Bacteriology Branch, Veterinary Sciences Division, Agri-Food and Biosciences Institute, Northern Ireland, Belfast, BT4 3SD UK; 3 Faculty of Management and Rural Development, University of Life Sciences King Mihai I from Timisoara, Timisoara, 300645 Romania; 4 Academy of Romanian Scientists, Ilfov Street, No. 3, Bucharest, 050044 Romania

**Keywords:** *Streptococcus agalactiae*, Tilapia (*Oreochromis niloticus*), Natural antimicrobials, Inflammation, Primary gut epithelial cells, Gut model

## Abstract

**Background:**

*Streptococcus agalactiae*, a Gram-positive bacterium, has emerged as an important pathogen for the aquaculture industry worldwide, due to its increased induced mortality rates in cultured fish. Developing interventions to cure or prevent infections based on natural alternatives to antibiotics has become a priority, however, given the absence of scientific evidence regarding their mode of action progress has been slow.

**Methods:**

In this study we aimed to investigate the effect of a mixture of organic acids (natural antimicrobials), AuraAqua (Aq), on the virulence of *S. agalactiae* using Tilapia gut primary epithelial cells and an in vitro Tilapia gut culture model. Our results show that Aq was able to reduce significantly, in vitro, the *S. agalactiae* levels of infection in Tilapia gut primary epithelial cells (TGP) when the MIC concentration of 0.125% was tested.

**Results and discussion:**

At bacterial level, Aq was able to downregulate bacterial capsule polysaccharide (CPS) gene expression, *capC*, resulting in a significant decrease in bacterial surface capsule production. The decrease in CPS production was also associated with a reduction in the pro-inflammatory IFNγ, IL1β, TNFα, SOD and CAT gene expression and H_2_O_2_ production in the presence of 0.125% Aq (*P* < 0.0001). The antimicrobial mixture also reduced the levels of *S. agalactiae* infection in an in vitro gut culture model and significantly reduced the IFNγ, IL1β, TNFα, SOD, CAT gene expression and H_2_O_2_ production in infected tissue. Moreover, genes involved in Tilapia resistance to *S. agalactiae* induced disease, MCP-8 and Duo-1, were also downregulated by Aq, as a consequence of reduced bacterial levels of infection.

**Conclusion:**

Conclusively, our study shows that mixtures of organic acids can be considered as potential alternative treatments to antibiotics and prevent *S. agalactiae* infection and inflammation in the Tilapia fish digestive tract.

## Background

Over the past thirty years, Nile Tilapia (*Oreochromis niloticus*) has ascended to the forefront of freshwater aquaculture, reaching a global market valuation of US $7.9 billion as of 2020, being cultivated in more than 120 of different nations [1, 2]. The cultivation of Nile Tilapia has transitioned from predominantly extensive farming practices to the proliferation of intensive, commercial aquaculture operations due to a rapid reproductive rates and pronounced solvability [3]. This shift has positioned Nile Tilapia farming as one of global aquaculture’s most rapidly expanding sectors [4]. Currently, Tilapia ranks as the third most prevalent species within aquaculture [2]. China, Indonesia, Malaysia, the Philippines, Thailand, Egypt, Honduras, Ecuador, and Costa Rica, including African countries have established themselves as leading Tilapia producers on the global market [5–7]. The volume of Tilapia production witnessed a substantial increase, expanding from 1.0 million tonnes in 2001 to 3.6 million tonnes by 2014 and 4.6 million tonnes in 2019 [2, 6]. Currently, Tilapia contributes approximately ≈ 4.8 million metric tons to worldwide food security, serving as a comparatively affordable protein source with premium meat quality [3, 4]. In 2017, Tilapia emerged as a significant agricultural product for Indonesia, which accounted for 18.43% of the total global output of 6,510,700 tons [5]. Within Indonesia, the Sumatra region is the preeminent Tilapia producer, contributing ≈ 48.0% of the nation’s output, followed by Java with ≈ 31.0% and Sulawesi at ≈ 11.50%.

*S*. *agalactiae*, an encapsulated Gram-positive bacterium, is traditionally classified by identifying distinct capsular polysaccharides, with ten capsular serotypes (Ia, Ib, and II to IX) recognized to date [7]. In marine organisms, three serotypes of GBS — specifically, Ia, Ib, and III — have been isolated from fish diagnosed with streptococcosis. However, in the context of Tilapia farms in Thailand, only two serotypes, Ia and III, have been documented [8]. *S*. *agalactiae* is encapsulated by a cotton-like layer that protects it. This capsule is present when the bacteria are free in the intestinal lumen, but notably, adherence to the epithelial surface leads to the capsule’s partial or complete disappearance, especially on the side facing away from the point of attachment [9]. This phenomenon, previously observed in in vitro TEM studies, indicates that *S*. *agalactiae* discards its capsule to bind to the epithelium [9]. The bacteria attach individually, in groups, or in chains, and can proliferate on the surface of enterocytes. GBS primarily binds and invades from the apical side of intestinal folds, dividing within enterocyte cytoplasm. Exiting the cells from the basolateral side, the bacteria enter the lamina propria and disseminate via microcirculation [9].

Managing streptococcosis in farmed Tilapia has predominantly relied on antibiotic treatments [6]. However, extensive antibiotic use has fostered antibacterial resistance, enabling the emergence and spread of resistant genes among fish, aquatic, and terrestrial pathogens. Additionally, prolonged antibiotic application can result in residues in fish products, potentially impacting human health. Since the EU banned antibiotics as growth and immunity enhancers in 2003, there has been a growing interest in natural products as alternative solutions for controlling fish diseases, highlighting the need for sustainable and health-conscious aquaculture practices. Natural antimicrobials, including essential oils, plant extracts and medicinal plants, probiotics, and combinations of organic acids with phytochemicals, have demonstrated in vitro and *ex-vivo* efficacy in diminishing bacterial pathogenicity [10, 11]. Their effectiveness is attributed to direct or indirect anti-virulence activities, which disrupt the mechanisms pathogens use to infect host organisms [12]. These natural substances offer a promising alternative to conventional antibiotics by targeting the virulence factors of pathogens without contributing to the development of antibiotic resistance, marking a pivotal shift towards more sustainable and less disruptive disease management strategies in aquaculture and other fields. In addition to mitigating bacterial pathogenicity, these natural antimicrobials can strengthen fish’s immune response and disease resilience and may even facilitate improved growth rates [2, 13–15].

The aim of this study was to bring further knowledge into our understanding of the mechanisms involved in the inhibition of *S. agalactiae* infection in Tilapia fish by a mixture of organic acids (natural antimicrobials). We aimed to understand their anti-inflammatory and antioxidant effects by looking at its impact on bacterial virulence factors using Tilapia gut primary epithelial cells and an in vitro Tilapia gut model of infection.

## Methods

### Preparation of Tilapia gut primary cells and S. agalactiae growth

The isolation of Tilapia gut primary cells (TGP) was performed as recently described [16]. Briefly, the gut tissue was sectioned and washed twice by centrifugation (5 min, 150×g). Five ml 0.25% trypsin at pH 7.4 at room temperature was added for 30–60 min and stirred on a magnetic stirrer, washed twice and cells were put into a 25 cm plastic culture flask with growth medium. Cells were further cultured at 28 °C in 24 well plates (Analab, Lisburn, UK) supplemented with 0.1% DMSO (Thermo-Fisher, UK) and 20% foetal bovine serum (FBS), 100 µg penicillin, 8% shrimp head extract, 6% salt solution, 20 ng epidermal growth factor (Sigma-Aldrich, Gillingham, UK) and 10 U/ml human recombinant interleukin 2 (Sigma-Aldrich, Gillingham, UK). Streptococcus *agalactiae* (*S. agalactiae*) was grown in TSAYE (Tryptone Soya Yeast Extract) at 37 °C. The natural antimicrobial mixture, AuraAqua (Aq), contains 5% maltodextrin, 1% sodium chloride, 42% citric acid, 18% sodium citrate, 10% silica, 12% malic acid, 9% citrus extract, and 3% olive extract (w/w). The raw materials were supplied by Bio-Science Nutrition Ireland. Experiments were carried out in triplicates (*n* = 3).

### Bacterial virulence assay

The TGP cells were grown in Dulbecco’s Modified Eagle Medium (DMEM) (Cell Biologics, USA) as described above and maintained in 75cm^2^ flasks (Sigma-Aldrich, Arklow, Ireland, SIAL0641) at 37 °C and 5% CO_2_. To test the effect of Aq on the ability of *S. agalactiae* to infect TGP cells, the assay was performed as previously described [17]. Briefly, the TGP cells (5.5 × 10^5^/well) were cultured for 24 h until 80–90% confluent in 6-well culture plates at pH 7.2. *S. agalactiae* bacteria were suspended in a DMEM medium at OD_600_ of ≈ 0.4 and added to FBS washed TGP cells at an MOI of 100. The AuraAqua (Aq) was also added in a 2 ml volume of DMEM containing a concentration of 0.125% Aq to the infected cells, gently centrifuged at 250×g for 5 min and incubated for a further 6 h. Additionally, in a second experiment the cells were treated with 0.125% Aq for 3 h prior to *S. agalactiae* infection. In a third experiment *S. agalactiae* bacteria were exposed for 3 h to 0.125% Aq and subsequently used to infected un-exposed TGP cells. Adherence was quantified by washing the infected monolayers with PBS followed by exposure to 0.1% Triton X100 in PBS for 15 min at 37 °C. Dilutions (10-fold) of infected or control wells were plated onto TSAYE agar and incubated for 2 days prior to enumeration, at 37 °C. All assays were performed in triplicate (*n* = 3) and on three separate days. Cytotoxicity of AuraAqua was determined as previously described [18] by utilizing the MTT assay (Sigma-Aldrich, Gillingham, England, UK).

### Minimum inhibitory (MIC) and minimum bactericidal (MBC) concentration against S. agalactiae

Both the MIC and the MBC were determined as previously described [19]. The two-fold tube dilution method was used to determine the lowest concentration of that inhibited bacterial growth (MIC) and the lowest concentration that induced bacterial death (MBC) was evaluated. AuraAqua was diluted (8–0.015625% v/v) in TSAYE broth and vortexed. Individual overnight bacterial cultures were harvested by centrifugation, washed twice in PBS, resuspended in TSAYE, and diluted to 1 × 10^6^ CFU/ml in TSAYE. Each tube was inoculated with 5 × 10^5^ CFU/ml of each bacterial culture (final concentration). Non-inoculated bijou (5 ml) tubes containing the same growth medium were used as negative controls, whilst MHB tubes without AuraAqua, were inoculated with individual bacterial cultures as positive controls. The *S. agalactiae* tubes were incubated at 37 °C for 48 h. Tubes that did not show visible growth were above the MIC. One hundred millilitres were taken from each tube for inoculation and then incubated at 37 °C for 24 h onto TSAYE agar at 37 °C for 48 h. The highest dilution of each antimicrobial with no microbial growth was considered as the MBC [51]. The antimicrobial mixture was tested using concentrations from 8 to 0.0078% (v/v) in three independent replicates repeated three times for each strain. To determine the sub-inhibitory concentrations used, all three pathogens were exposed to different concentrations of the antimicrobial mixture. The highest concentrations of antimicrobial that showed no effect on survivability and no growth inhibition (same growth kinetics as the control) were used in subsequent experiments.

### Measurement of IL-1β, TNF-α and IFN-γ gene expression in TGP cells and infected gut tissue in vitro

At 6 h post infection the impact of 0.125% Aq was also measured by its impact on IL-1β, TNF-α and IFN-γ genes expression. To perform measurements in the infected tissue, in vitro, the gut tissue was disrupted by sonication for 60 s (4X) at 4 °C (in ice) in 1% saline solution followed by centrifugation at 2500 rpm at 4 °C for 5 min. We have also quantified the relative expression of MCP-8 and Duo-1, in the in vitro gut model, in the presence or absence of 0.125% Aq. For the control the β-actin gene was used as reference. The RNeasy Plus Mini Kit (Qiagen, Manchester, UK) kit was used for RNA isolation. Reverse-transcribed RNA was obtained by using the Transcriptor First Strand cDNA Synthesis Kit (Roche, Dublin, Ireland). cDNA (1 µl) was added to duplicate wells with 7 µl of DNase-free water, 10 µl of Taqman Fast Advanced master mix (Applied Biosystems) and 1 µl of each of 2 Taqman gene expression assays (Applied Biosystems). Samples were incubated at 50 °C for 2 min followed by 95 °C for 20 s then cycled 40 times at 95 °C for 3s and 60 °C for 30 s in a LightCycler 96 (Roche). The 2^–ΔΔCT^ method was used to analyse the relative expression (fold changes), calculated relative to the control group. All primers are included in Table 1.

**Table 1:** List of primers used in this study

Gene name	Primers	Reference
IL-1β	F - tgctgagcacagaattccag	[20]
	R - gctgtggagaagaaccaagc	
TNF-α	F - gaggtcggcgtgccaaga	
	R - tggtttccgtccacagcgt	
IFN-γ	F - tgaccacatcgttcagagca	
	R - ggcgacctttagcctttgt	
MCP-8	F - cgggttagctgttggcattgt	[21]
	R - aagcaagcagagaaaaccacttca	
Duo-1	F–tggctggatggggaacaactaa	[22]
	R–ccagaggaccaccagaatcac	
*capC*	F - catccagagcggaataaagc	[23]
	R - gtgttatgcgcatctgaacc	
β-actin	F - tgacccagatcatgttcgagac	
	R - gtggtggtgaaggagtagcc	

### S. agalactiae capsule polysaccharide (CPS) detection and capC gene expression

Capsule polysaccharide was prepared from bacteria co-cultured with TGP cells which were pretreated with 0.125% AuraAqua and from bacteria directly exposed to 0.125% AuraAqua for 6 h. Additionally, capsules were extracted from bacteria co-cultured with TGP cells and 0.125% Aq or in the absence of Aq. As controls capsules from liquid grown and plate grown *S. agalactiae* were also extracted. Bacteria were harvested by centrifugation and suspended in 100 µl of lysis buffer containing 31.25 mM Tris-HCl (pH 6.8), 4% sodium dodecyl sulphate, 0.025% bromophenol blue, and 20% glycerol. After heating to 100 °C for 5 min, 5 µl of 20 mg/ml proteinase K was added to the solution, and the tubes were incubated for 1 h at 50 °C. The samples were separated on NuPageNovex 12% bis-Tris gels (Invitrogen, United Kingdom). Following electrophoresis, gels were stained with an Alcian blue (Sigma Aldrich, United Kingdom) solution containing 0.1% Alcian blue, 40% ethanol, and 5% acetic acid. For *capC* gene expression analysis the total RNA was isolated from bacteria exposed to 0.125% AuraAqua at a concentration of 0.125% for 3 h by using the RNeasy®Plus Mini Kit (Qiagen, United Kingdom). The RNA was reverse transcribed using Transcriptor First Strand cDNA Synthesis Kit (Roche, United Kingdom) according to the manufacturer’s protocol. The mRNA levels were determined by quantitative RT-PCR using QuantiNovaSYBR® Green PCR Kit (Qiagen, United Kingdom) on a LightCycler 96 (Roche, United Kingdom). The primers used (Invitrogen, United Kingdom) for *capC* are presented in Table 1. The conditions for genes rRNA 16 S consisted of incubating for 10 min at 95 °C followed by 45 cycles of 95 °C for 10 s, 55 °C for 30 s, and 72 °C for 10 s. A total of 5 µl of SYBR Green master mixture was used in each reaction along with 0.5 µl of 10 µM primer mixture, 3 µl of molecular grade water, and 1 µl of DNA sample. Green master mixture was used in each reaction along with 0.8 µl of 20 µM primer mixture, 7.4 µl of molecular grade water, and 1 µl of DNA sample. Relative quantity of the mRNA was calculated using the ΔCt method. The 16 S gene was used as an endogenous control.

### Measurement of SOD and CAT activity in S. agalactiae infected TGP cells and the gut tissue model

The activity of superoxide dismutase (SOD) and catalase (CAT) was measured as previously described [24]. Briefly, the TGP cells only, the infected TGP cells or infected TGP cells in the presence of 0.125% Aq were washed with PBS and disrupted with Trypsin PBS solution. The cells were then centrifuged for 10 min at 1400×g and resuspended in lysis buffer containing protease inhibitors. After 30 min of incubation on ice, the extraction mixture was centrifuged at 12,000× g at 4 °C for 30 min. SOD activity was determined using a commercially available SOD colorimetric activity kit (Thermo Fisher, UK) and CAT by using a catalase activity kit (Abcam, Trumpington, England, UK, ab83464). The procedures were followed as per manufacturer instructions. To perform measurement in the infected ex vivo, the gut tissue was disrupted by sonication for 60 s (4X) at 4 °C (in ice) in 1% saline solution followed by centrifugation at 2500 rpm at 4 °C for 5 min. The supernatant was used to determine the superoxide dismutase (SOD) and catalase (CAT).

### Quantification of capsular polysaccharides attached to the cell surface

To quantify the CPS production levels the alkaline extraction of CPS from bacteria was performed as previously described with some modifications [25]. In short, *S. agalactiae* was grown in 20 ml of THB at 37 °C for 10 h. Bacterial cells were harvested by centrifugation for 15 min at 3,220xg at 4 °C and resuspended in PBS, 0.8 N NaOH; and incubated at 37 °C for 36 h. After two washes with distilled H_2_O, the CPS extract was recovered by resuspension in water. A similar protocol was applied by harvesting bacteria from plate grown *S. agalactiae* (fully covered plate). The amount of CPS present in the extract was estimated by measuring the sialic acid content using the colorimetric resorcinol-hydrochloric acid method by mixing 120 µl of extract with 380 µl of water and 500 µl of resorcinol solution. After boiling for 20 min the absorbance was measured at 564 nm.

### In vitro culture model of Tilapia intestinal cells

An in vitro culture model of Tilapia intestine was established as previously described [26] and later modified for infections assay [27]. Intestinal sections were transferred to Dulbecco’s modified Eagle’s medium (DMEM) containing 10% bovine serum albumin (BSA) and further incubated at 28 °C, 5% CO_2_ and 80% relative humidity for 3 h to assess tissue integrity. Sterility tests were performed in brain heart infusion (BHI) media. Selected gut sections were inoculated with 10^7^*S. agalactiae* diluted in tissue culture media for 6 h. The infected gut sections were also used for IL-1β, TNF-α and IFN-γ gene expression and measurement of catalase (CAT) and superoxide dismutase activity with the respective methodologies described above. For *S. agalactiae* enumeration, the infected or un-infected intestinal sections were homogenized in PBS, and plated at serial dilutions onto TSAYE agar and incubated for 2 days prior to enumeration, at 37 °C. The results are presented as CFU/ml homogenised tissue. The experiments are graphically presented in Fig. 4A1 and A1.

### Extracellular hydrogen peroxide (H_2_O_2_) measurements in infected TGP cells and in the Tilapia gut model

The amount of hydrogen peroxide release by the infected TGP cells of in the Tilapia gut model was measured as previously described [28]. An H_2_O_2_ Amplex® UltraRed /HRP (Thermo Fischer Scientific, UK) kit was used according to the manufacturers’ instructions. Briefly, the lysis buffer or culture media (50 ml) were mixed with the Amplex® UltraRed /HRP (Thermo Fischer Scientific, UK) reagent and with the horseradish peroxidase resulting in a red fluorescent oxidation product. Fluorescence was determined at 530 nm excitation and 590 nm emission using a fluorescence microplate reader (FLUOstar Omega, BMG Labtech). The concentrations of H_2_O_2_ were calculated using standard curves. All experiments were repeated three times.

### Statistical analysis

Statistical analyses were performed using GraphPad software. Data were represented as mean ± SD. *P*-values < 0.05 were considered statistically significant following estimations using the Student *t* was used.

## Results

### S. agalactiae growth and virulence in Tilapia gut primary epithelial cells (TGP) in the presence of Aq

**Fig. 1:**
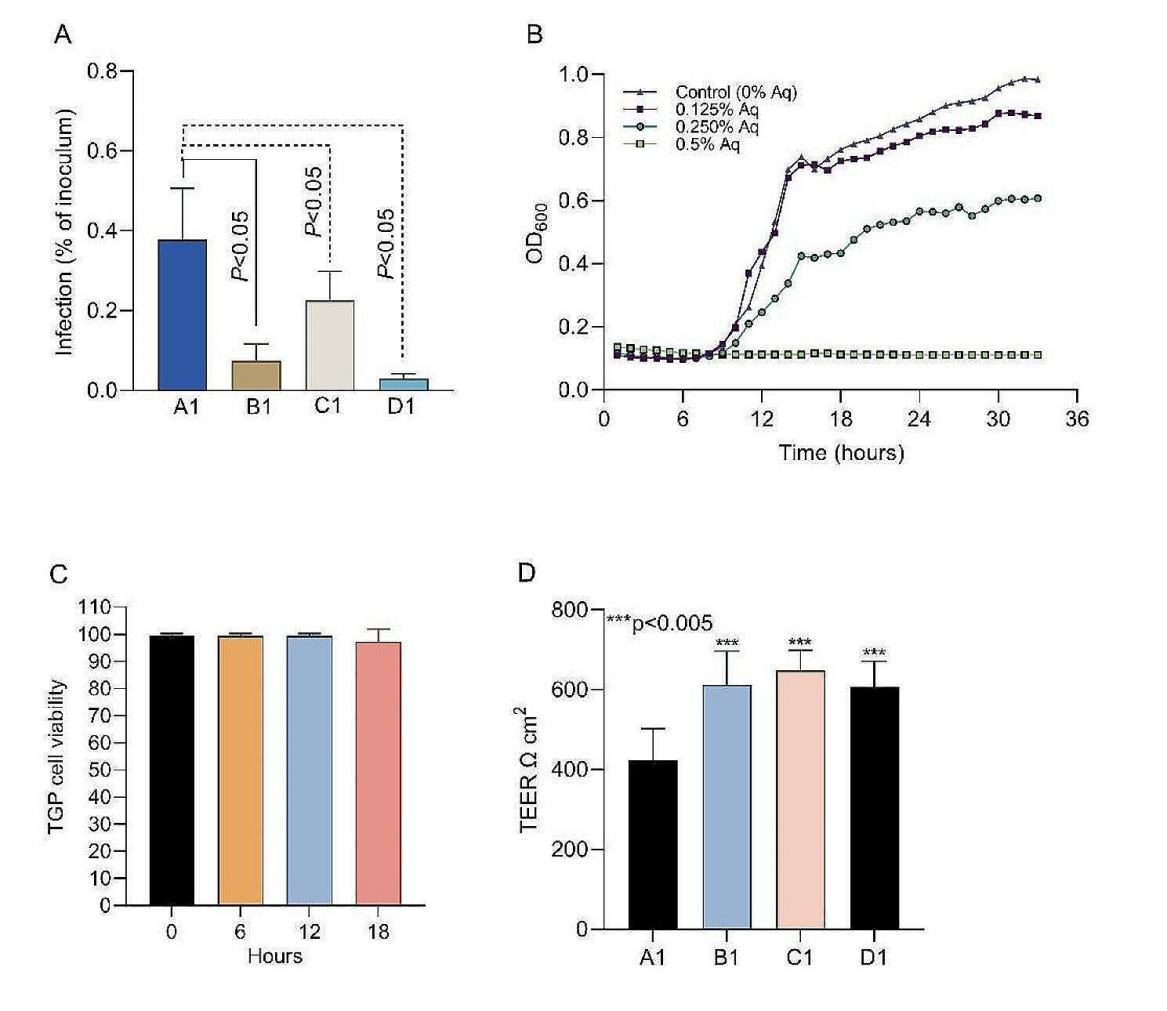
The impact of Aq on the growth and infective efficacy of *S. agalactiae*. Panel A shows the *S. agalactiae* infection of TGP cells (A1 – control infection of TGP cells with *S. agalactiae*; B1 – *S. agalactiae* pre-treated with 0.125% Aq prior to infection; C1 – TGP cells infected in the presence of 0.125% Aq; D1 - TGP cells were pre-treated with 0.125% Aq prior to infection). Panel B – *S. agalactiae* growth curve. Panel C – TGP cell viability in the presence of 0.125% Aq. Panel D – Transepithelial resistance (TEER). All experiments were performed triplicate, and each measurement was performed from three separate samples. A *P* value less than 0.05 was considered significant

First, we aimed to establish the MIC and the MBC concentrations at which the natural antimicrobial mixture (AuraAqua) inhibits or blocks *S. agalactiae* growth. Antimicrobial activity was detected at 0.125% (MIC) with the minimum bactericidal concentration being established at 0.50% (MBC). The MIC concentration of 0.125% Aq was selected to further assess its anti-infective role in Tilapia gut primary cells (TGP). Our results show that at prior exposure of bacteria to 0.125% Aq significantly prevented (*P* < 0.05) *S. agalatiae* infection of TGP cells (Fig. 1A-B1) compared to un-exposed bacteria (Fig. 1A-A1). Similar results (*P* < 0.05) were observed when 0.125% Aq was present in the media during infection with un-exposed *S. agalactiae* (Fig. 1A-C1) or when the TGP cells were pre-treated with 0.125% Aq prior to infection (Fig. 1A-D1). Figure 1B presents the growth abilities of *S. agalactiae* in the presence of 0.5%, 0.250%, and 0.125% Aq. In Fig. 1C our data suggests that the antimicrobial mixture itself had no impact on the viability of the TGP cells. Moreover, the presence of the Aq has a clear strengthening impact on the TGP cellular junctions, as indicated in Fig. 1D. In conclusion, these results reflect the positive impact of Aq in preventing bacterial invasion of TGP cells and in strengthening the cellular tight junction without affecting cellular integrity.

### The impact of Aq on S. agalactiae capsule polysaccharide (CPS) expression and production during infection of TGP cells

**Fig. 2:**
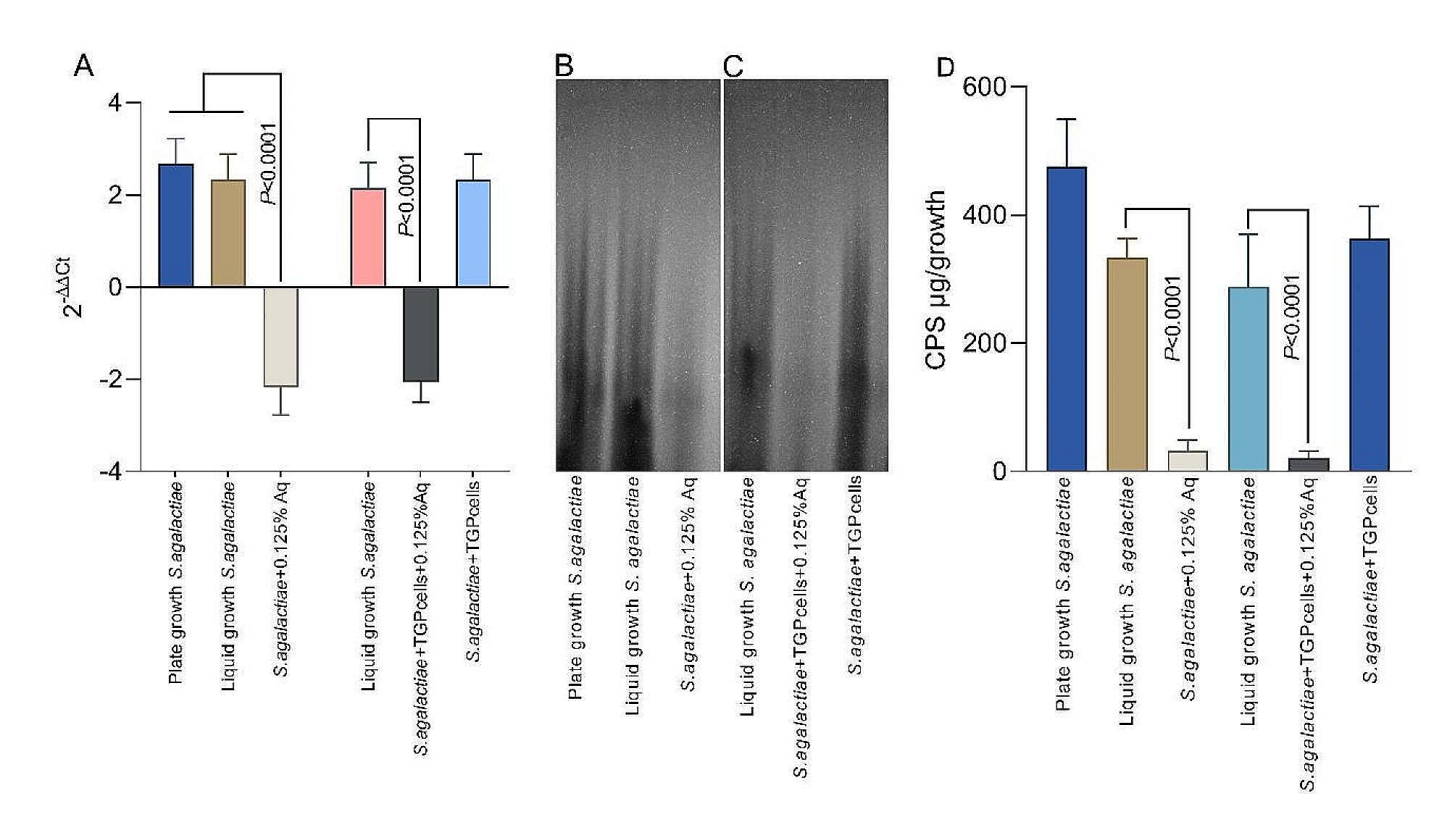
*S. agalactiae* CPS production during infection of TGP cells in the presence of 0.125% Aq. Panel A – *capC* expression during growth and in co-culture in the presence of 0.125% Aq. Panels B and C – Alcian blue staining of bacterial capsules during growth and co-culture. Panel D – CPS production quantification during growth and co-culture. All experiments were performed triplicate, and each measurement was performed from three separate samples. A *P* value less than 0.05 was considered significant

Based on the observation that *S. agalactiae* pre-exposure to 0.125% Aq reduces the bacterium ability to infect TGP cells, we have next investigated the impact on the bacterial CPS production as one of its significant virulence factors. First, we show that the capsular polysaccharide synthesis gene, *capC*, was significantly downregulated (*P* < 0.0001) when *S. agalactiae* was grown in the presence 0.125% Aq (Fig. 2A) leading to a reduction in CPS detection by Alcian blue staining (Fig. 2B) and in CPS production (Fig. 2D). In co-culture with TGP cells and in the presence of 0.125% Aq a similar reduction was observed (Fig. 2A and C), reduction which was further confirmed by a decrease in CPS production (Fig. 2D). During co-culture with TGP cells, but in the absence of Aq, the levels of CPS production by *S. agalactiae* were not affected (Fig. 2A, C and D). These results suggest that Aq is indeed responsible for CPS depletion in *S. agalactiae* during infection, leading to a reduced ability to attach or infect gut epithelial cells (TGP).

### Pro-inflammatory responses in TGP cells infected with S. agalactiae in the presence of 0.125% Aq

**Fig. 3:**
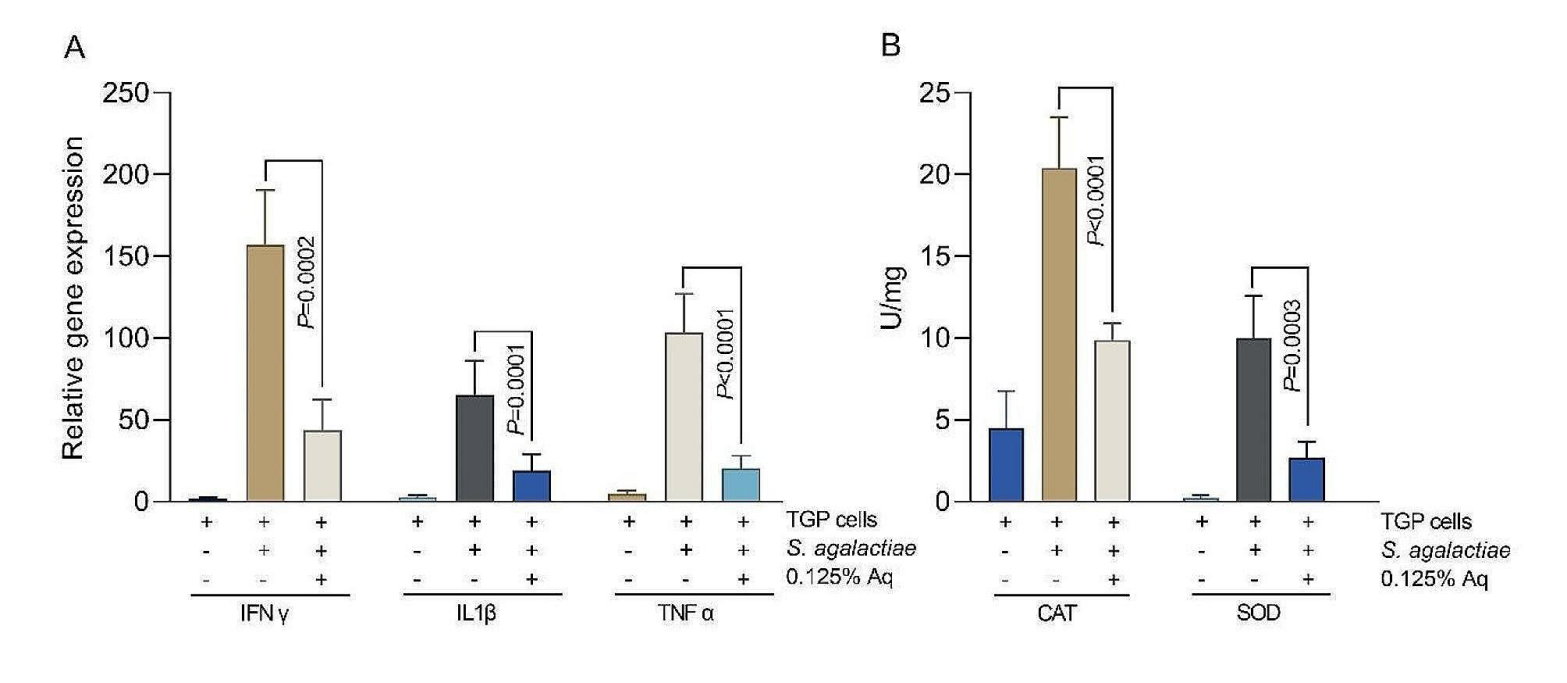
Pro-inflammatory genes expression (IFNγ, IL1β, TNFα), catalase (CAT) and superoxide dismutase (SOD) in infected TGP cells in the presence of 0.125% Aq. Data are expressed as 2^−ΔΔCt^; values are the mean of three test replicates relative to the reference gene 16 S RNA ribosomal subunit. *P* values are indicated on the graphs

Next, we aimed to investigate if following *S. agalactiae* capsule reduction, upon exposure to 0.125% Aq, the bacterium is still able to induce a pro-inflammatory response in the infected TGP cells. First, as shown in Fig. 3A *S. agalactiae* can induce a significant increase in IFNγ, IL1β and TNFα gene expression in the infected TGP cells. This expression was significantly inhibited by the presence of 0.125% Aq during infection, suggesting that the inability of bacteria to infect the TGP cells results in reduced cellular inflammation. Secondly, it has resulted in reduced oxidative stress as indicated by the reduced levels of catalase (CAT) (*P* < 0.0001) and superoxide dismutase (SOD) (*P* = 0.0003) produced by the TGP cells during infection and in the presence of 0.125% Aq.

### Ex vivo infection of Tilapia gut tissue in the presence of 0.125% Aq (gut model)

**Fig. 4:**
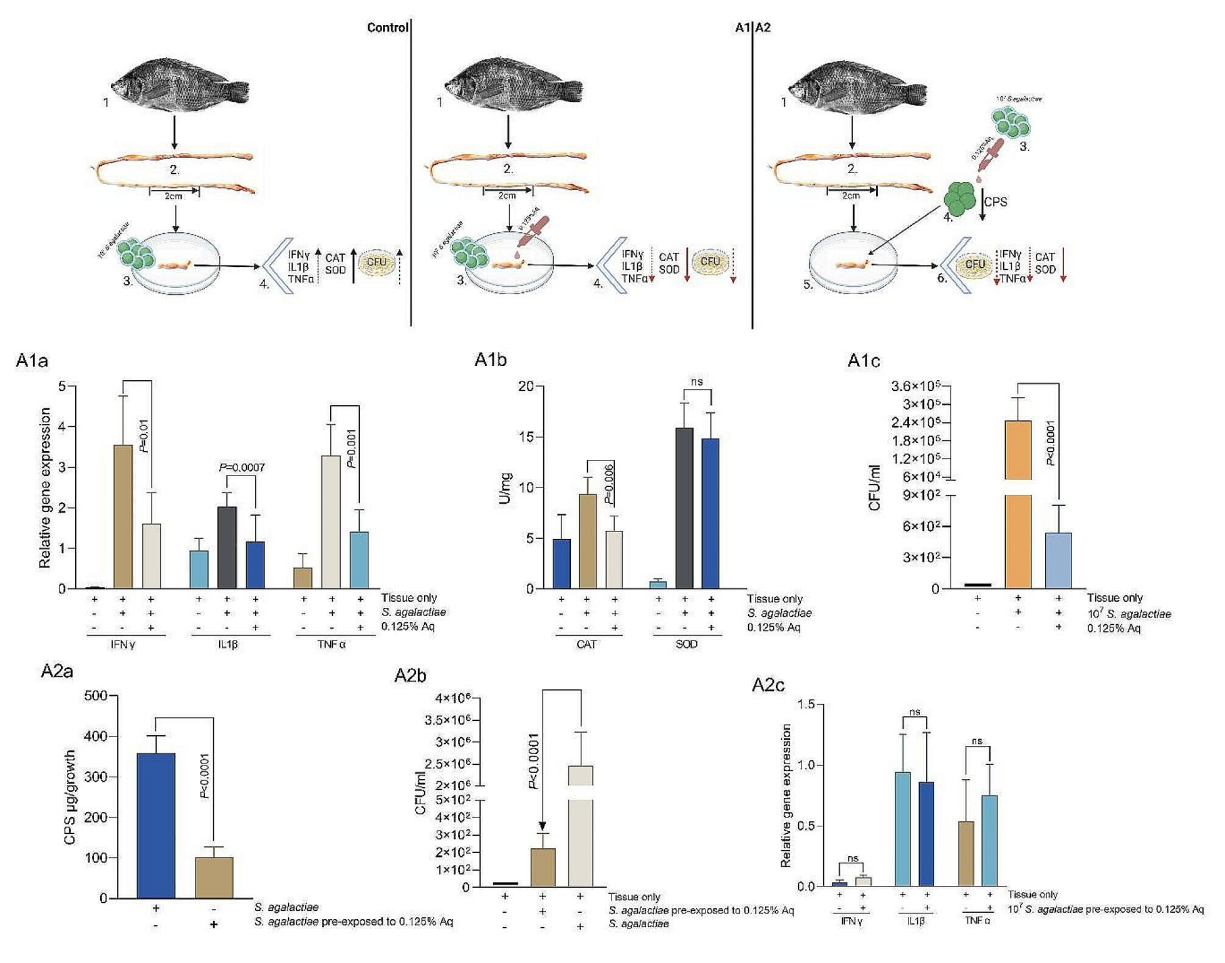
The impact of Aq on the pro-inflammatory gene expression (IFNγ, IL1β, TNFα) (Panel A); catalase (CAT), superoxide dismutase (SOD) (Panel B) and bacterial infection levels (Panel C) in infected gut tissue ex vivo. Gene expression data are expressed as 2^−ΔΔCt^; values are the mean of three test replicates relative to the reference gene 16 S RNA ribosomal subunit. The infection data is presented as CFU/ml lysate. The Student’s *t*-test was used to statistically compare the effect of AuraAqua. *P* values are indicated on the graphs. Experimental designed was produced using Biorender.com

To confirm the in vitro anti-inflammatory results, we have set up an ex vivo organ culture experiment using intestinal sections as described in material and methods. The first experiment (Fig. 4A1) was designed to test the impact of 0.125% Aq during infection with *S. agalactiae* without pre-exposure to Aq. In this instance, the proinflammatory responses in the infected tissue was significantly reduced in the presence of 0.125% Aq as reflected in the IFNγ (*P* = 0.01). IL1β (*P* = 0.0007) and TNFα (*P* = 0.001) gene expression levels (Fig. 4A1a). An antioxidative effect was emphasized through reduced CAT levels (*P* < 0.006) and SOD, however, for SOD the decrease was not statistically significant (Fig. 4A1b). The antioxidant and anti-inflammatory effect were also associated with reduced levels of *S. agalactiae* in the infected gut tissue (*P* < 0.0001) (Fig. 4A1c). The second experiment (Fig. 4A2) was designed to test if decapsulated *S. agalactiae*, following pre-exposure to Aq, are still able to infect the gut tissue and cause an inflammatory response. As indicated in Fig. 4A2a, after 3 h of exposure the bacterium was significantly decapsulated (*P* < 0.0001). As a result, the levels of infection were only of 10^2^, similar to the levels in experiment A1 (Fig. 4A2b). These low levels of infection did not cause a significant inflammatory response and are comparable to the levels of IFNγ, IL1β and TNFα in un-infected tissue (Fig. 4A2c). Our results confirm that Aq can prevent *S. agalactiae* infection ex vivo, avoid inflammatory and oxidative events and reconfirms the role of Aq in infection through its decapsulation effect on *S. agalactiae*.

### MCP-8 and Duo-1 gene expression in Tilapia gut tissue in the presence of 0.125% Aq

**Fig. 5:**
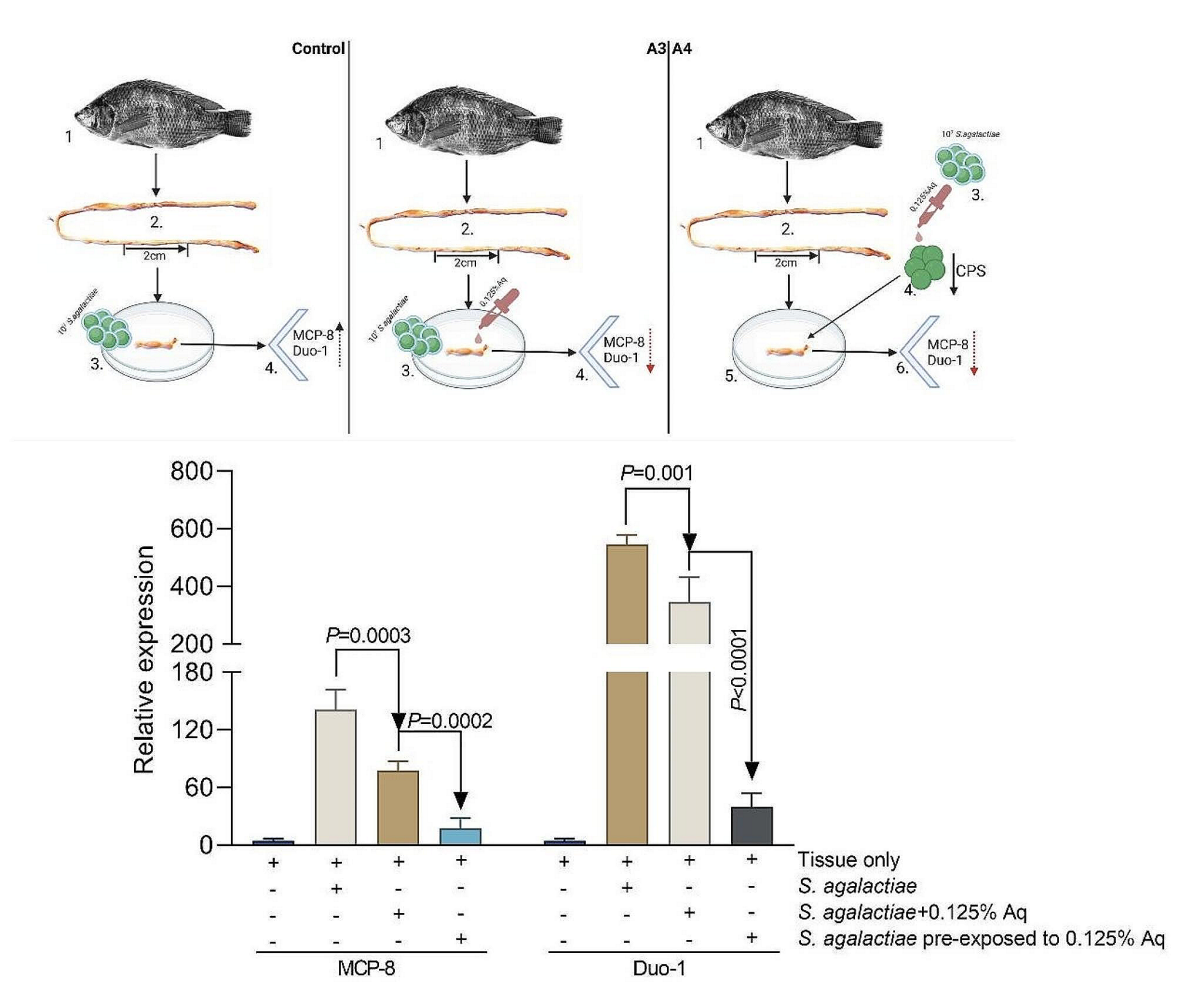
The impact of Aq on the expression of MCP-8 and Duo-1 in the in vitro Tilapia gut model. Panel A – experimental diagram. Panel B – MCP-8 and Duo-1 gene expression in the *S. agalactiae* infected tissue in the presence of 0.125% Aq or in tissue infected with Aq pre-treated *S. agalactiae*. Gene expression data are expressed as 2^−ΔΔCt^; values are the mean of three test replicates relative to the reference gene 16 S RNA ribosomal subunit. The infection data is presented as CFU/ml lysate. The Student’s *t*-test was used to statistically compare the effect of AuraAqua. *P* values are indicated on the graphs. Experimental designed was produced using Biorender.com

We have also used the in vitro Tilapia gut model to investigate the impact of Aq (0.125%) on the expression of *S. agalactiae* genes involved in disease resistance. The experimental design (Fig. 5A) included a control experiment in which Tilapia gut tissue was infected with 10^7^*S. agalactiae*, for 6 h, in the absence of Aq. In the second experiment (Fig. 5A-A3) the excised gut section was infected with 10^7^*S. agalactiae*, for 6 h, in the presence of 0.125% Aq. In the last experiment 10^7^*S. agalactiae* was exposed for 3 h to 0.125% Aq and subsequently used to infect the excised tissue for 6 h (Fig. 5A-A4). Our results indicate a significant increase in MCP-8 and Duo-2 gene expression in the control experiment infected with 10^7^*S. agalactiae* and in the absence of Aq. However, a significant decreasing trend, in the expression of both MCP-8 and Duo-1, was detected in experiments A3 and A4, likely caused by the already described reduction in the infection rate. These results clearly emphasize the link between *S. agalactiae* infection and Tilapia response to disease and links the outcome to the presence/absence of the antimicrobial mixture (Aq).

### The impact of Aq on H_2_O_2_ production in S. agalactiae infected gut tissue or TGP cells

**Fig. 6:**
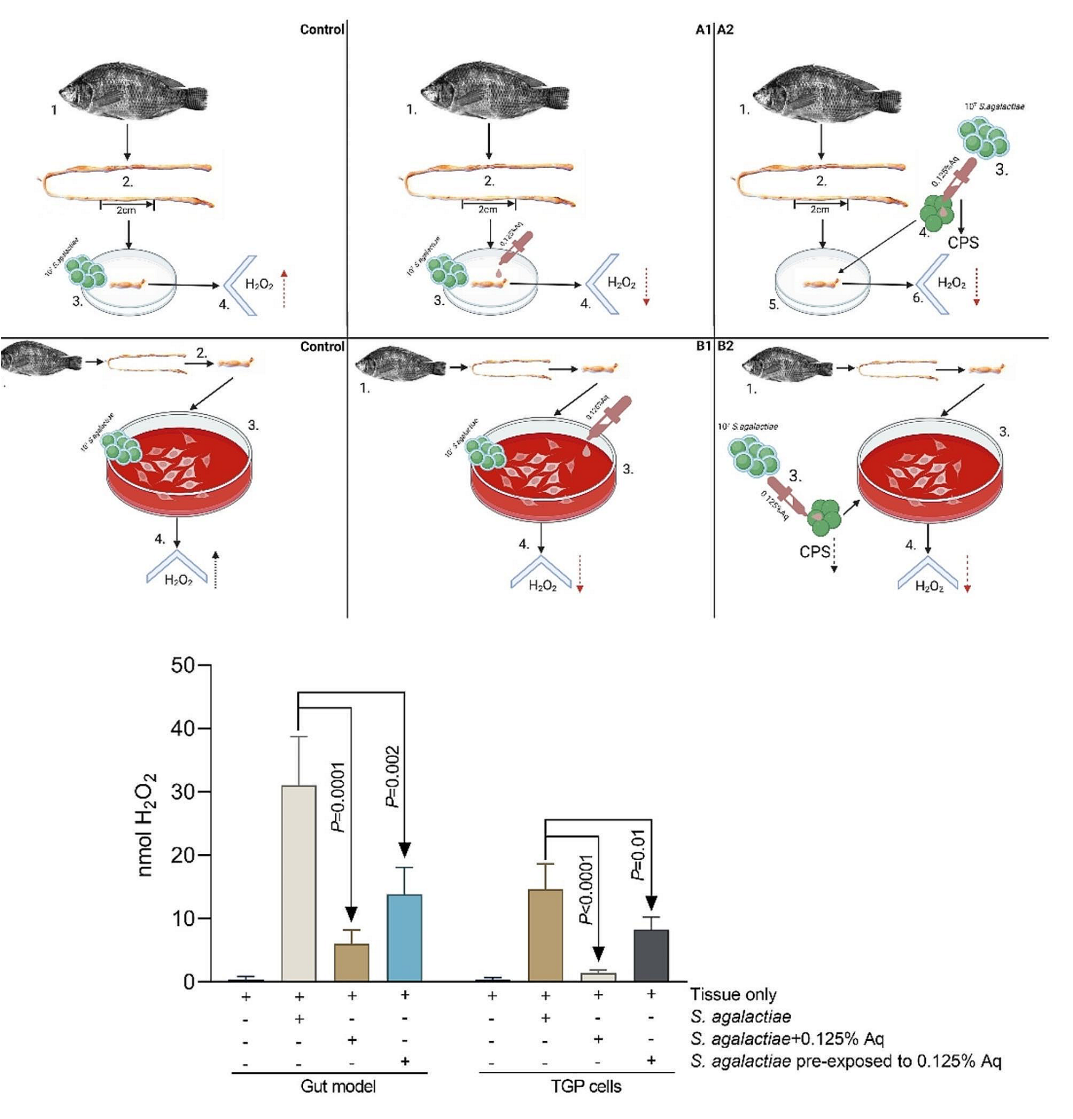
The impact of 0.125% Aq on the levels of H_2_O_2_ produced by the *S. agalactiae* infected gut tissue or the TGP cells. Panel A and B experimental design. Panel C - The extracellular levels of H_2_O_2_ released by the infected gut tissue and TGP cells. The significance levels are indicated on the graph. Data are presented as means (SD) of triplicate independent samples and experiments. Experimental designed was produced using Biorender.com

To follow up on the anti-inflammatory effect we have set up a trial experiment to investigate the impact on the ability of the infected gut tissue or TGP cells to produce and release hydrogen peroxide (H_2_O_2_). The experimental diagram is presented in Fig. 6A and B. Trial 6A included a control experiment in which Tilapia gut tissue was infected with 10^7^*S. agalactiae*, for 6 h, in the absence of Aq. In the second experiment (Fig. 6A-A1) the excised gut section was infected with 10^7^*S. agalactiae*, for 6 h, in the presence of 0.125% Aq. In the last experiment 10^7^*S. agalactiae* were exposed for 3 h to 0.125% Aq and subsequently used to infect the excised tissue for 6 h (Fig. 6A-A2). Trial 6B followed a similar design with the exception that in this case the impact on H_2_O_2_ was measured in TGP cells. As indicated in Fig. 6C in the gut model the levels of H_2_O_2_ produced were significantly reduced when the tissue was infected with *S. agalactiae* and in the presence of 0.0125% Aq (*P* = 0.0001) or when the tissue was infected with *S. agalactiae* pre-exposed to 0.125% Aq (*P* = 0.002). The levels of H_2_O_2_ produced by the TGP cells followed by a similar pattern in all three scenarios and resulted in a significant decrease in the presence of 0.125% Aq. Our conclusive observation is that the decline in H_2_O_2_ produced and released by the Tilapia gut tissue or primary cells is a consequence of reduced *S. agalactiae* infection levels in the presence of Aq.

## Discussion

Notoriously, *Streptococcosis*, a condition caused by infection with Group B *Streptococcus* (GBS), represents a problematic challenge within the Tilapia aquaculture and other fish sectors [29, 30]. In 2011, the disease lead to an economic loss of approximately $40 million within China’s Tilapia industry, attributed to its high morbidity and mortality rates, which may surge to 80% during outbreak episodes [29]. Streptococcal bacteria, recognized as facultative anaerobic pathogens, adversely affect both cultivated and wild Tilapia populations [11]. Species such as *Streptococcus agalactiae*, *Streptococcus iniae* and *Streptococcus shiloi*, belonging to the *Streptococcaceae* family, prevail in warm climates and are primary culprits behind septicemia and meningoencephalitis in Tilapia and across other species [11]. Clinical manifestations of Streptococcal infections in these fish are characterized by aberrant behaviour and erratic swimming patterns, loss of appetite, exophthalmos with ocular haemorrhage, corneal opacity, curvature of the spinal cord, muscular stiffness, and haemorrhagic lesions at the fin bases and meningitis [2, 4, 5].

The natural antimicrobial mixture used in this study (Aq) was able to significantly reduce the ability of *S. agalactiae* to infect Tilapia gut primary epithelial cells (TGP) especially when cells or bacteria are pre-exposed to 0.125% Aq before infection. The presence of Aq during the infection assay, without pre-exposure, also significantly impeded infection. This was the first indication that the antimicrobial mixture can induce changes in bacteria and the host cells with a detrimental impact on the infection levels. Such changes can include depletion of bacterial capsule polysaccharide (CPS), a major virulence factor, leading to a significantly reduced capacity to cause in vivo infections in Tilapia fish [30]. This increased virulence boost, in capsulated *S. agalactiae* is justified by the bacterium ability to evade host’s defence mechanisms by interfering with phagocytic cells [31]. Controversially, *S. agalactiae* bacterial capsules can also impede adhesion to Tilapia intestine, however, adhesion can be further regulated by the acidic milieu in encapsulated strains [32]. Encouragingly, our study shows that loss of capsule, caused by exposure of *S. agalactiae* to a mixture of natural antimicrobials, leads to decreased infection levels and inflammatory responses both in vitro and in a Tilapia gut model of infection. The capsular reduction effect of natural antimicrobials was previously demonstrated in *S. aureus* by downregulating the expression of the *capC* gene, responsible for the production of the capsular polysaccharide synthesis enzyme (Cap8C) by nisin [23].

By using an in vitro gut model, herein, we show that the expression of the pro-inflammatory cytokines IFNγ, IL1β, and TNFα were significantly upregulated when Tilapia gut tissue was infected with *S. agalactiae*, alongside catalase (CAT), superoxide dismutase (SOD) as indicators of increased oxidative stress. The presence Aq had a potent anti-inflammatory effect and significantly reduced the expression of IFNγ, IL1β, and TNFα together with CAT and SOD. The impact of Aq on bacterial decapsulation, as observed in vitro with TGP cells, was mimicked in the in vitro organ model, as our data indicates a significantly reduced ability of decapsulated *S. agalactiae* to infect or cause an immune response. The immune response induced by the decapsulated *S. agalactiae* was increased but not significantly different when compared to the levels detected in the uninfected tissue. Secondly, *S. agalactiae* infected Tilapia intestines are known to express a cell protease 8 (MCP-8) [21] and a duodenase-1 enzyme [22], both being involved in resistance to disease though immune regulation. In our study we have also observed that upon infection *S. agalactiae* was also able to upregulate both genes in an in vitro Tilapia gut culture model and we show that the natural antimicrobial mixture (Aq) reduced their expression during infection by blocking attachment of *S. agalactiae* to the intestinal tissue. Critically, we show that this reduced interaction is achieved through reduced CPS production in *S. agalactiae*. Overall, we demonstrated that the natural antimicrobial mixture (Aq) can block *S. agalactiae* infection of Tilapia gut cells and tissue through induced bacterial CPS expression.

Through their complex biological mechanisms, natural antimicrobials can significantly mitigate the adverse effects of gene expression related to inflammation, apoptosis, and immunity [33] and repair the hepatic and splenic structural damage caused by the bacterial infection [33]. The novel mechanistic insights described in this manuscript together with the data already present in the literature suggest that natural antimicrobials are a promising strategy for enhancing disease resilience in aquaculture through dietary modifications.

## Conclusions

Currently preventing infections based on natural alternatives (organic acids) is difficult to approach in the absence of solid scientific evidence on their mode of action. Our results show that Aq was able to significantly reduce *S. agalactiae* infection of Tilapia gut primary epithelial cells (TGP) and was able to downregulate bacterial capsule polysaccharide (CPS) expression, reduce the pro-inflammatory IFNγ, IL1β, TNFα, SOD and CAT gene expression and H_2_O_2_ production. The antimicrobial mixture also decreased the levels of *S. agalactiae* infection in an in vitro gut infection model. Conclusively, our work shows that mixtures of organic acids can be efficiently used as interventions and prevent *S. agalactiae* infection and inflammation in Tilapia fish.

## Data Availability

The datasets used and analysed during the current study are available from the corresponding author on reasonable request.

## Abbreviations

TGP: Tilapia Gut Primary Cells
Aq: AuraAqua
Duo: 1–Duodenase–1

## References

[1] Delphino M, Joshi R, Alvarez AT (2022). Economic appraisal of using genetics to control Streptococcus agalactiae in Nile tilapia under cage and pond farming system in Malaysia. Sci Rep.

[2] Wang B, Thompson KD, Wangkahart E, Yamkasem J, Bondad-Reantaso MG, Tattiyapong P (2023). Strategies to enhance tilapia immunity to improve their health in aquaculture. Reviews Aquaculture.

[3] Sutthi N, Wangkahart E, Panase P, Karirat T, Deeseenthum S, Ma NL (2023). Dietary Administration effects of Exopolysaccharide produced by Bacillus tequilensis PS21 using Riceberry Broken Rice, and soybean meal on growth performance, immunity, and resistance to Streptococcus agalactiae of Nile tilapia (Oreochromis niloticus). Animals.

[4] Ghetas H, Neiana A, Khalil R, AM H, Khallaf M (2021). Streptococcus agalactiae isolation and characterization in Nile tilapia (Oreochromis niloticus) with histopathological studies. J Curr Veterinary Res.

[5] Suhermanto A, Sukenda S, Zairin M Jr, Lusiastuti AM, Nuryati S. Characterization of Streptococcus agalactiae bacterium isolated from tilapia (Oreochromis niloticus) culture in Indonesia. Aquaculture, Aquarium, Conservation & Legislation. 2019;12(3):756 – 66.

[6] Na-Phatthalung P, Chusri S, Suanyuk N, Voravuthikunchai SP (2017). In vitro and in vivo assessments of Rhodomyrtus tomentosa leaf extract as an alternative anti-streptococcal agent in Nile tilapia (Oreochromis niloticus L). J Med Microbiol.

[7] Delannoy CMJ, Samai H, Labrie L (2021). Streptococcus agalactiae serotype IV in farmed tilapia. Aquaculture.

[8] Kayansamruaj P, Pirarat N, Katagiri T, Hirono I, Rodkhum C (2014). Molecular characterization and virulence gene profiling of pathogenic Streptococcus agalactiae populations from tilapia (Oreochromis sp.) farms in Thailand. J Vet Diagn Invest.

[9] Vásquez-Machado G, Barato-Gómez P, Iregui-Castro C (2019). Morphological characterization of the adherence and invasion of Streptococcus agalactiae to the intestinal mucosa of tilapia Oreochromis sp.: an in vitro model. J Fish Dis.

[10] Balta I, Linton M, Pinkerton L, Kelly C, Ward P, Stef L (2021). The effect of natural antimicrobials on the Campylobacter coli T6SS+/– during in vitro infection assays and on their ability to adhere to chicken skin and carcasses. Int J Food Microbiol.

[11] Nami Y, Kahieshesfandiari M, Lornezhad G, Kiani A, Elieh-Ali-Komi D, Jafari M et al. Administration of microencapsulated Enterococcus faecium ABRIINW.N7 with fructo-oligosaccharides and fenugreek on the mortality of tilapia challenged with Streptococcus agalactiae. Front Vet Sci. 2022;9. 10.3389/fvets.2022.938380 PMC9376237 35978708

[12] Pinkerton L, Linton M, Kelly C, Ward P, Gradisteanu Pircalabioru G, Pet I (2019). Attenuation of Vibrio parahaemolyticus virulence factors by a mixture of natural antimicrobials. Microorganisms.

[13] Melo-Bolívar JF, Ruiz Pardo RY, Hume ME, Nisbet DJ, Rodríguez-Villamizar F, Alzate JF (2019). Establishment and characterization of a competitive exclusion bacterial culture derived from Nile tilapia (Oreochromis niloticus) gut microbiomes showing antibacterial activity against pathogenic Streptococcus agalactiae. PLoS ONE.

[14] Brum A, Cardoso L, Chagas EC, Chaves FCM, Mouriño JLP, Martins ML (2018). Histological changes in Nile tilapia fed essential oils of clove basil and ginger after challenge with Streptococcus agalactiae. Aquaculture.

[15] Van Doan H, Hoseinifar SH, Hung TQ, Lumsangkul C, Jaturasitha S, Ehab E-H (2020). Dietary inclusion of chestnut (Castanea sativa) polyphenols to Nile tilapia reared in biofloc technology: impacts on growth, immunity, and disease resistance against Streptococcus agalactiae. Fish Shellfish Immunol.

[16] Butucel E, Balta I, McCleery D, Marcu A, Stef D, Pet I et al. The Prebiotic Effect of an Organic Acid mixture on Faecalibacterium prausnitzii metabolism and its anti-pathogenic role against Vibrio parahaemolyticus in shrimp. Biology (Basel). 2022;12(1). 10.3390/biology12010057 PMC9855566 36671749

[17] Butucel E, Balta I, Bundurus IA, Popescu CA, Iancu T, Venig A (2023). Natural antimicrobials promote the anti-oxidative inhibition of COX-2 mediated inflammatory response in primary oral cells infected with Staphylococcus aureus, Streptococcus pyogenes and Enterococcus faecalis. Antioxidants.

[18] Butucel E, Balta I, McCleery D, Popescu CA, Iancu T, Pet I et al. The Effect Citrox BCL on Legionella pneumophila mechanisms of Biofilm formation, oxidative stress and virulence. Antioxid (Basel). 2022;11(11). 10.3390/antiox11112186 PMC9686968 36358558

[19] Balta I, Marcu A, Linton M, Kelly C, Gundogdu O, Stef L (2021). Mixtures of natural antimicrobials can reduce Campylobacter jejuni, Salmonella enterica and Clostridium perfringens infections and cellular inflammatory response in MDCK cells. Gut Pathog.

[20] Panase A, Thirabunyanon M, Promya J, Chitmanat C (2022). Influences of Bacillus subtilis and fructooligosaccharide on growth performances, immune responses, and disease resistance of Nile tilapia, Oreochromis niloticus. Front Vet Sci.

[21] Fu GH, Wan ZY, Xia JH, Liu F, Liu XJ, Yue GH (2014). The MCP-8 gene and its possible association with resistance to Streptococcus agalactiae in tilapia. Fish Shellfish Immunol.

[22] Shen Y, Fu GH, Liu F, Yue GH (2015). Characterization of the duodenase-1 gene and its associations with resistance to Streptococuus agalactiae in hybrid tilapia (Oreochromis spp). Fish Shellfish Immunol.

[23] Zhao X, Meng R, Shi C, Liu Z, Huang Y, Zhao Z (2016). Analysis of the gene expression profile of Staphylococcus aureus treated with nisin. Food Control.

[24] Wang M., Lu M., Zhang C., Wu X., Chen J., Lv W., Sun T., Qiu H., Huang S. Oxidative stress modulates the expression of tolllike receptor 3 during respiratory syncytial virus infection in human lung epithelial A549 cells. Mol. Med. Rep. 2018;18:1867–1877. 10.3892/mmr.2018.9089. 10.3892/mmr.2018.9089 29845280

[25] Toniolo C, Balducci E, Romano MR, Proietti D, Ferlenghi I, Grandi G (2015). Streptococcus agalactiae capsule polymer length and attachment is determined by the proteins CpsABCD. J Biol Chem.

[26] Barato P, Martins ER, Vasquez GM, Ramirez M, Melo-Cristino J, Martinez N (2016). Capsule impairs efficient adherence of Streptococcus agalactiae to intestinal epithelium in tilapias Oreochromis Sp. Microb Pathog.

[27] Vasquez-Machado G, Barato-Gomez P, Iregui-Castro C (2019). Morphological characterization of the adherence and invasion of Streptococcus agalactiae to the intestinal mucosa of tilapia Oreochromis sp.: an in vitro model. J Fish Dis.

[28] Corcionivoschi N, Alvarez LA, Sharp TH, Strengert M, Alemka A, Mantell J. Mucosal reactive oxygen species decrease virulence by disrupting Campylobacter jejuni phosphotyrosine signaling. Cell Host Microbe. 2012;12. 10.1016/j.chom.2012.05.018 PMC3749511 22817987

[29] Zhang Z (2021). Research advances on Tilapia Streptococcosis. Pathogens.

[30] Zhang D, Ke X, Liu Z, Cao J, Su Y, Lu M (2019). Capsular polysaccharide of Streptococcus agalactiae is an essential virulence factor for infection in Nile tilapia (Oreochromis niloticus Linn). J Fish Dis.

[31] Leghari A, Lakho SA, Khand FM, Bhutto KR, Lone SQ, Aleem MT (2023). Molecular epidemiology, characterization of virulence factors and antibiotic resistance profile of Streptococcus agalactiae isolated from dairy farms in China and Pakistan. J Integr Agric.

[32] Barato P, Martins ER, Vasquez GM, Ramirez M, Melo-Cristino J, Martínez N (2016). Capsule impairs efficient adherence of Streptococcus agalactiae to intestinal epithelium in tilapias Oreochromis Sp. Microb Pathog.

[33] Wang F, Xian X-R, Guo W-L, Zhong Z-H, Wang S-F, Cai Y (2020). Baicalin attenuates Streptococcus agalactiae virulence and protects tilapia (Oreochromis niloticus) from group B streptococcal infection. Aquaculture.

